# Internal Strengths for Adverse Life Events

**DOI:** 10.3390/bs14080665

**Published:** 2024-08-01

**Authors:** Jian Zhao, Elaine Chapman, Stephen Houghton

**Affiliations:** Graduate School of Education, University of Western Australia, Perth, WA 6009, Australia; jian.zhao@uwa.edu.au (J.Z.); stephen.houghton@uwa.edu.au (S.H.)

**Keywords:** internal strengths, COVID-19, Chinese students, mental health, adverse life events

## Abstract

In this study, a brief measure of four internal attributes found to protect against the impact of adverse life events on mental health was developed and validated. In addition, profiles of internal strengths that significantly predict mental health outcomes in young Chinese adults were identified. The results of exploratory and confirmatory factor analyses on data from 831 Chinese university students supported the proposed four-factor model of the ISALES. Participants in the current study fell into two clusters, with one cluster being higher than the other in all four internal strengths, with the former cluster demonstrating better overall mental health than those in the latter cluster. The ISALES is a promising instrument for use in clinical settings and may be used to identify individuals who are more ‘at risk’ of developing poor mental health in the aftermath of a negative life event. The use of the ISALES may permit tailored interventions and timely support to be provided to individuals within clinical settings.

## 1. Introduction

Research has confirmed that adverse life events increase the risk of developing mental health problems such as anxiety and depression [1,2,3]. This research has highlighted attributes that may protect individuals from the impact of such events, including high problem-solving self-efficacy, internal locus of control, the ability to deal with uncertainty, and mindfulness exhibited by the individual in such situations [4,5,6,7]. All of these factors are recognised to be key contributors to the resilience of individuals in confronting negative life events or their ability to successfully adapt to challenging life experiences [8].

The American Psychological Association [8] argued further that an individual’s ability to adapt to challenging experiences would depend upon various internal strengths possessed by the individual, including their cognitive profile; the external sources of social support they could draw upon; and their use of particular kinds of coping strategies. These three types of factors may be related to one another in a time-sequenced manner. For example, individuals who have significant internal strengths (such as high levels of self-efficacy) are likely to view adverse events as somewhat less threatening than those who lack these internal strengths. Such individuals may, as a result, be better placed to access and utilise available sources of support (e.g., friendship networks) and adopt more adaptive coping strategies than those who become overwhelmed immediately in the face of an adverse event. In response to the feeling of being overwhelmed by the event, the latter individual might be more likely to adopt shorter-term, maladaptive coping strategies (e.g., withdrawal) as a reactionary response. Thus, sound internal strengths may be an important antecedent in overall adaptive responses to adverse events.

The salutogenic model proposed by Antonovsky offers a broader perspective on these internal strengths by focusing on the origins of health rather than the prevention of disease [9]. Antonovsky’s Sense of Coherence (SOC) framework suggests that individuals who perceive their life as comprehensible, manageable, and meaningful are more likely to maintain health and well-being [9]. This model emphasises the importance of generalised resistance resources (GRRs) that facilitate successful coping with stressors inherent in life, thereby fostering a sense of coherence. Factors discussed in this paper—problem-solving self-efficacy, internal locus of control, ability to deal with uncertainties and mindfulness—are key determinants of whether individuals perceive their lives as comprehensible, manageable, and meaningful, aligning closely with the GRRs that promote effective coping with stressors.

Various instruments for measuring the resilience levels of individuals have already appeared in the literature. The Sense of Coherence scale (SOC) developed by Antonovsky [10,11] provides a valuable tool for measuring how individuals perceive their lives in terms of comprehensibility, manageability, and meaningfulness. The SOC scale has been used across different populations and cultural contexts and seems to be a reliable, valid instrument to measure how individuals manage stressful situations despite some inconsistencies in the factorial structure [10,12]. However, while the SOC scale is beneficial for understanding general perceptions of life coherence, it may not capture the specific cognitive and psychological attributes that directly contribute to resilience in the face of adverse life events.

In a systematic review conducted by Windle et al. [13], 15 self-report instruments designed to measure resilience for general and clinical use were considered. Windle et al. [13] noted that none had been evaluated comprehensively in terms of psychometric properties (e.g., criterion validity, content validity, internal consistency). Certain instruments were also deemed to be questionable in terms of their conceptual and theoretical inadequacy, for example, the Adolescent Resilience Scale and the Resilience Scale [14,15]. Furthermore, 7 of the 15 instruments were developed for use with children and adolescents, and therefore may not be suitable for use with adults [13].

A further problem with most of the existing instruments reviewed by Windle et al. [13] is that they may be too broad in what they attempt to measure. For example, the Connor–Davidson Resilience Scale (CD-RISC) developed by Connor and Davidson [16] was designed to measure five factors (i.e., personal competence, high standards, and tenacity; trust in one’s instincts, tolerance of negative affect, and strengthening effects of stress; positive acceptance of change and secure relationships; control; and spiritual influences). Similarly, the Youth Resiliency: Assessing Developmental Strengths Questionnaire developed by Donnon and Hammond [17] aims to assess a broader range of 10 factors including “Family”, “Community”, “Peers”, “Work (commitment to learning)”, “School (culture)”, “Social Sensitivity”, “Cultural Sensitivity”, “Self-concept”, “Empowerment”, and “Self-control” (p. 972). Owing to this, the content of the instruments may lack the specificity needed to achieve sound construct validity.

As noted, based on the APA definition, at least three types of factors may be important in determining an individual’s level of resilience (i.e., “the ways in which individuals view and engage with the world”, “the availability and quality of social resources”, and “specific coping strategies”). Each of these may, however, be considered a construct in its own right. Therefore, instruments designed to assess all of these attributes may in effect measure none of them comprehensively.

### 1.1. Theoretical Background

Given that the internal strengths discussed are likely to function as an important precursor to either an adaptive or maladaptive response to an adverse life event, it may be useful for clinicians to have a brief instrument to measure these antecedent attributes alone. The current study aimed to develop a brief instrument to measure the first type of factor identified by the American Psychological Association—that is, the internal strengths that affect how individuals view and engage with adverse events or experiences. A review of the literature indicated that four such attributes (mindfulness, ability to deal with uncertainty, problem-solving self-efficacy, and internal locus of control) are likely to be important in this respect.

### 1.2. Mindfulness

Mindfulness, defined by Brown and Ryan [18] (p. 822) as “the state of being attentive to and aware of what is taking place in the present”, has now emerged as a significant concept in mental health research [19] (p. 230). In particular, mindfulness has been found to reduce and prevent ruminative and depressogenic thinking, thereby promoting the resilience levels of individuals following adverse life events [20,21]. Individuals who adopt a mindful orientation in their everyday lives are therefore more likely to be able to respond in an adaptive way to negative experiences.

### 1.3. Ability to Deal with Uncertainty

Uncertainty is characteristic of many adverse life events, and many individuals have been found to respond to uncertainty in a negative way [22]. One early study [23] found that uncertainty can act as a powerful stressor. People who are incapable of dealing with uncertainty are therefore likely to respond more poorly to many adverse life events [24]. In line with this proposition, people who lack the skills to deal with uncertainty have been found to perceive uncertainty as a threat and to experience more negative effects on mental health in response to any events that are associated with such uncertainty [25,26].

### 1.4. Problem-Solving Self-Efficacy

Self-efficacy has been defined broadly as people’s beliefs in their capabilities to exercise influence over events that affect their lives [27]. Problem-solving self-efficacy (i.e., an individual’s perceptions of their capacity to solve problems or handle difficult situations) may protect people from the negative psychological effects of adverse life events because those with high self-efficacy may appraise stressful situations as challenges to be addressed, rather than as threatening and insurmountable obstacles [28]. People with a strong sense of their problem-solving abilities therefore may be better able to adapt to stressful situations that are brought about by adverse life events.

### 1.5. Internal Locus of Control

Rotter [29] described locus of control as an individual’s tendency to attribute positive or negative experiences either to their own actions or to external factors such as luck. People who believe that they cannot do much to change the nature of their experiences are said to have more of an external locus of control, while those who believe that their experiences reflect their own actions or personal characteristics are said to have more of an internal locus of control [30]. People who tend to have an internal locus of control may consider themselves capable of dealing with challenging situations and may thus choose to take a more active approach to dealing with these situations. As a result of this, individuals with an internal locus of control may be better able to adapt and ‘bounce back’ in the face of adversity.

### 1.6. Proposed Interactions between Strengths

At a minimum, to respond effectively to problems created by adverse events and ‘bounce back’ from these events, an individual initially needs to achieve the following:

(i) Remain engaged with what is happening presently, irrespective of whether this is a positive or a negative experience (i.e., exhibit mindfulness in confronting the experiences precipitated by these events).
(ii) Be able to tolerate uncertainty, given that most adverse events are accompanied by higher levels of uncertainty than positive events.
(iii) Have confidence in his/her own ability to solve any specific problems that are created by the adverse event.
(iv) Perceive dealing with the event to be within his or her own control, rather than under the control of external factors (e.g., luck) alone.

All of these factors are likely to affect how people view and engage with adverse experiences or events, which will then influence how they cope with these experiences and events. As a result, these factors may be key moderators of the longer-term effects of adverse events on mental health.

### 1.7. Objective

The first aim of this study was to develop and validate a brief instrument (the Internal Strengths for Adverse Life Events Scale, or ISALES) to measure self-reported levels of problem-solving self-efficacy, locus of control, ability to deal with uncertainty, and mindfulness in adults. Therefore, the ISALES deals primarily with the cognitive, internal strengths that individuals may access in response to adverse life events. The instrument does not deal with other aspects of resilience such as access to social support. The second aim was to create profiles of internal strengths based on scores from these four subscales and investigate whether individuals with different strength profiles differed significantly in terms of their mental health and wellbeing.

## 2. Materials and Methods

### 2.1. Participants

The participants were 828 Chinese students studying in Australian universities (*n* = 96) or Chinese universities (*n* = 732). For the validation, these students were divided randomly into two groups. Group A comprised *n* = 414 (224 male, 190 female) participants, with ages ranging from 18 to 49 years (M = 22.52, SD = 4.23). Group B comprised *n* = 414 (229 male, 184 female, 1 other) participants, whose ages ranged from 18 to 48 years (M = 22.53, SD = 4.35).

### 2.2. Instruments

The World Health Organization Well-Being Index (WHO-5), first presented by the WHO Regional Office in Europe in 1998, is a 5-item unidimensional scale measuring subjective mental well-being [31]. Respondents rate each item (e.g., “I have felt calm and relaxed) on a five-point scale (from “At no time = 0” to “All of the time = 5”) based on their experiences over the past two weeks. Higher scores indicate higher levels of wellbeing on the scale. The WHO-5 has been demonstrated to have strong validity for measuring mental health outcomes in various previous evaluations [32].

The 12-Item Depression, Anxiety, and Stress Scale (DASS-12) is a brief self-report questionnaire designed to measure indicators of negative mental health [33,34]. The DASS-12 comprises three subscales (Depression, Anxiety, and Stress), each of which includes four items (e.g., “I found it hard to wind down”). Respondents rate each item on a 4-point scale ranging from 0 (did not apply to me at all) to 3 (applied to me very much). Scores for each subscale are summed, with higher scores indicating more negative states on that specific subscale. The DASS-12 has been demonstrated to have sound psychometric properties in previous studies [34,35].

The 16-item ISALES was designed to measure four internal strengths: (a) mindfulness; (b) ability to deal with uncertainty; (c) problem-solving self-efficacy; and (d) locus of control. Each item is presented as a 7-point bipolar statement, and respondents need to rate each item using a scale ranging from −3 to +3, with higher scores indicating higher levels of that internal strength. The ISALES was developed in both Chinese and English (see the full instrument in Table 1). Participants in the current study completed the Chinese version.

### 2.3. Data Collection and Analysis

Data were collected online via Qualtrics. The study was first approved by the authors’ University Human Ethics Committee before being completed by students. Over 1000 students provided online consent to participate in the study, but several did not provide complete responses. A final sample of 828 responses was retained for analysis after removing incomplete responses and clearly disengaged answers (i.e., responses in which the same rating was given for all items). No financial incentives were offered for participation in the study.

Exploratory factor analyses (EFAs), and confirmatory factor analyses (CFAs) were conducted to evaluate the internal structure of the ISALES. Cronbach’s αs were also generated for each factor to provide evidence on the reliability of the instrument.

A two-step cluster analysis was then performed to determine whether individuals with different levels of internal strength exhibited different profiles. People with different profiles of internal strengths were also compared to determine whether they exhibited different levels of mental health (based on the WHO-5 and DASS-12) using a *t*-test.

The EFA, Cronbach’s αs, correlations, and t-test were all conducted through IBM SPSS V27, and the CFAs were conducted through LISREL V10.0.

## 3. Results

The validity of the ISALES was evaluated in terms of four of the five types of validity evidence described by Messick [36,37] and the 2014 Standards for Educational and Psychological Testing [38]. The one form of evidence not presented in this paper relates to the consequences of test use, which cannot be evaluated until the instrument has been used over a longer period of time.

### 3.1. Validity Evidence Based on Test Content

Items in the ISALES were developed based on an extensive review of existing instruments available in either English or Chinese [18,29,39,40,41,42,43,44,45,46]. The relevant literature search involved multiple academic databases, including PubMed, PsycINFO, and Google Scholar, using keywords such as “adverse life events”/“negative life events” + “mental health” + “mindfulness”/“ability to deal with uncertainties”/“problem-solving self-efficacy”/“locus of control”. We included peer-reviewed articles that provided empirical evidence and theoretical discussions on the protective factors against adverse/negative life events and that are written in English or Chinese. We also reviewed existing resilience scales and measures to ensure that the identified attributes were well-supported by previous research. This review process ensured a solid theoretical foundation for selecting the constructs measured by the ISALES.

This process was used to ensure that the content of the instrument aligned with the constructs targeted in the instrument. All items are presented as bipolar statements. The bipolar statement scale is a specific type of rating scale proposed and used extensively in previous research by the second author [47] which requires respondents to place themselves on a continuum between two opposing statements. The bipolar scale has the advantage of measuring not only the direction but also the intensity of the respondent’s opinion on the target concept [48]. To provide further assurance of the content validity of the ISALES, after all items were developed, the instrument was sent to two experts with more than 30 years of clinical experience in the mental health field for feedback. Any items that were deemed to be unclear or that were questioned in terms of their relationship to the intended constructs were then revised by the authors. The first author, who is qualified in translation between Chinese and English, then checked each item to ensure that the two versions conveyed the same meanings regardless of the language used.

### 3.2. Validity Evidence Based on Response Processes

Cognitive interviews were conducted with four Chinese university students on a one-on-one basis. In each interview, the participant was invited to read the survey carefully and mark the items that were unclear or confusing to them. This process helped to identify potential misinterpretations of the items that would occur in the completion of the survey and to ensure that the response processes associated with completing the instrument aligned with the intentions of the developers. Based on the four Chinese students’ feedback, further revisions and adjustments were made to three items in both versions.

### 3.3. Validity Evidence Based on Internal Structure

To evaluate the internal structure of the ISALES, EFAs and CFAs were performed with the data from Groups A and B, respectively. Cronbach’s αs for two groups were also computed to evaluate the internal consistency of each factor. First, data from Group A were factor-analysed using Maximum Likelihood extraction to explore the number of dimensions that were tenable for the ISALES. As the components were expected to be relatively independent of one another, these were rotated to approximate simple structure using the Varimax approach. With a case-to-item ratio of 25.88, the sample size was large enough to yield reliable estimates. Rotated loadings are presented in Table 2. The loadings suggested a four-factor model, which accounted for 67.50% of the total item variance. The scree plot also confirmed the four-factor model.

Pearson correlation analyses between the factor scores were conducted to explore the independence among the highlighted factors. The results in Table 3 show that the correlation coefficients between the factors range from 0.54 to 0.66, all significant at *p* < 0.01 level (2-tailed). These results indicate moderate correlations between the factors. While the factors are correlated, the moderate strength of these correlations supports the notion that they represent related but distinct dimensions of internal strengths. This finding is consistent with the theoretical framework that suggests these factors, while interconnected, contribute uniquely to resilience in the face of adverse life events.

Therefore, the moderate inter-factor correlations suggest that while there is some overlap, the factors maintain a degree of independence, justifying their inclusion as separate constructs within the ISALES. This supports the validity of the ISALES in measuring distinct aspects of internal strengths that protect mental health during stressful situations.

For the CFAs on data from Group B, two CFA models were established. Again, a case-to-item ratio of 25.88 indicated that the sample size was large enough to yield reliable estimates. The χ^2^s across the two models were then compared to evaluate whether the model fit statistics for the two models were significantly different. The first was a one-factor model, in which all 16 items loaded onto a single factor; the second was the four-factor model suggested by the EFA results. The fit indices for the two models tested are shown in Table 4. The four-factor model was clearly better fitting than the one factor, given the significant increase in χ^2^ from the four- to the one-factor model, Δχ^2^ (6) = 1039.07, *p* < 0.001.

**Figure 1:**
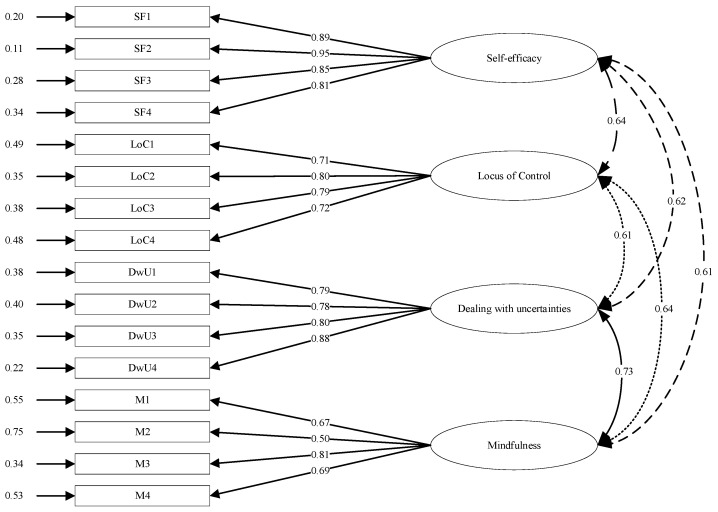
Path diagram for the ISALES based on data of Group B.

Other fit indices also confirmed the sound fit of the four-factor model. More specifically, the χ^2^/df was 3.97, with values ≤ 5 indicating an acceptable model fit. The sound fit of the four-factor model was also confirmed by other fit indices [49,50], including a Non-Normed Fit Index (NNFI) of 0.92 (values ≥ 0.90 indicating acceptable model fit), a Comparative Fit Index (CFI) of 0.93 (values ≥ 0.90 indicating acceptable model fit) and a standardised Root Mean Residual (SRMR) of 0.05 (values ≤ 0.08 indicating acceptable model fit). Coefficients for the paths between the latent factors and corresponding items can be seen in Figure 1. Cronbach’s αs based on data from Group A ranged from 0.82 to 0.94, while those for Group B ranged from 0.77 to 0.93. These results also confirm that the instrument exhibited acceptable levels of internal consistency.

### 3.4. Validity Evidence Based on Relationships with Mental Health

Previous research has suggested that self-efficacy [7], internal locus of control [4], and mindfulness [51] are all positively related to mental health. Uncertainty may bring stress, which has been found to be negatively associated with mental health [52]. As such, the ability to deal with uncertainty has also been related theoretically to individuals’ mental health. To further explore the validity of the ISALES in terms of its relationships with other variables, a two-step cluster analysis based on z-scores from the four ISALES measures was performed to create internal strength profiles. This analysis indicated an optimal solution of two clusters, with one cluster being high in all four internal strengths (cluster 1, *n* = 337) and the other being low in all four strengths (cluster 2, *n* = 491). The mean and standard deviation (SD) for clusters 1 and 2 are listed in Table 5.

A one-way MANOVA was then performed to explore whether participants from the two clusters differed in their scores on the WHO-5 and the three subscales of DASS-12. The MANOVA indicated a significant multivariate effect for cluster membership, λ = 0.88, F(4, 820) = 29.42, *p* < 0.001, and all univariate ANOVAs on the four individual subscales were also significant using a Bonferroni-adjusted α level of 0.0125, Fs(1, 823) > 20.90, ps < 0.001, partial η^2^ > 0.03. More specifically, participants of cluster 1 (*n* = 337) had higher levels of well-being and lower levels of depression, stress and anxiety than cluster 2 (*n* = 491). This affirms that the profiles based on the four ISALES measures were significantly related to mental health against the backdrop of COVID-19.

## 4. Discussion

The ISALES is designed to measure the internal strengths that adults may draw upon when encountering a negative life event, with a particular focus on aspects of resilience that fall within the cognitive domain. This evaluation provided evidence to support the validity of the instrument in terms of its content, response processes, internal structure, and relations with other variables. The internal consistency of the subscales within the instrument was also found to be acceptable.

The factor loadings for LoC1 (“I feel that what happens in my life depends a lot on chance and luck”) and M1 (“Usually when I’m engaged in an activity I often find myself thinking about other things”) were less than 0.6. However, these two items were retained in the scale due to their theoretical importance, contribution to construct validity, empirical considerations, and practical implications. These items capture critical dimensions of the internal locus of control and mindfulness, ensuring a comprehensive assessment of these constructs.

One of the key strengths of the ISALES is its brevity, with only 4 items per variable and a total of 16 items to assess four variables, making it concise and efficient. This brevity is particularly useful for quick screening in both clinical and research settings, which allows for rapid assessment without compromising the depth and accuracy of the measurement. The short length also reduces respondent burden, increasing the likelihood of high completion rates and reliable responses.

The ISALES may be useful for measuring the internal strengths that protect individuals’ mental health in times of stress. This instrument would, for example, be useful in clinical settings, particularly in designing and implementing interventions tailored to individual needs. By assessing individuals‘ problem-solving self-efficacy, locus of control, ability to deal with uncertainty, and mindfulness, clinicians can create detailed profiles of internal strengths for each client and use them to guide targeted therapeutic approaches, which may lead to more personalised and effective interventions. For example, clients who exhibit low levels of problem-solving self-efficacy and low levels of ability in dealing with uncertainty might benefit from cognitive–behavioural strategies that help enhance problem-solving skills and tolerance for uncertainty. Interventions such as problem-solving therapy (PST) [53] could be adopted to improve their confidence in dealing with negative life events, while mindfulness programmes [54] can help them develop a more accepting and present-focused attitude, preventing anxious worry and negative thinking that can increase emotional distress. Moreover, clients with low levels of internal locus of control might struggle with feelings of depression or anxiety [55], and cognitive-based interventions such attribution retraining may be useful in this regard.

Incorporating the ISALES into routine clinical assessments may also help to identify individuals who are more mentally ‘at risk’ in stressful situations or following adverse life events. By regularly monitoring these internal strengths, clinicians can proactively address potential vulnerabilities and provide timely support. This preventive approach would not only assist in mitigating the impact of adverse events but may also foster long-term resilience and well-being.

The current study inevitably has some limitations. First, data on the ISALES were collected at a single timepoint. As a result, evidence related to the consequences of using the instrument as stipulated by [36] are unavailable for the instrument at present. Second, although the initial psychometric data from Chinese university students on the ISALES in the present study are encouraging and promising, we acknowledge that our sampling technique, primarily involving young adult students recruited from universities, may limit the generalisability of our findings. This convenience sampling approach could introduce bias and may not fully represent the broader population, including older adults and individuals of different ages or from different educational or cultural backgrounds. Future research should aim to include more diverse samples or extend this work by examining gender invariance to ensure that the validity of the instrument remains invariant across different contexts or different gender groups. Third, while the development of the ISALES was based on an extensive review of the relevant literature, a multimethod approach including interviews may be warranted to provide more detailed information about the internal strengths of individuals when confronting an adverse event. Moreover, normative data would provide benchmarks that can be used to compare individual scores and determine where they stand in relation to a broader, diverse population. Future studies should develop these norms across various demographic groups to enhance the ISALES’s utility. Therefore, while the present results are promising, further work evaluating the validity of the ISALES is warranted to ensure that the generality of the instrument is upheld across contexts.

## Figures and Tables

**Table 1:** Item statements for the ISALES.

Label	Low-Scoring Statement (Scored 1 at Endpoint) 代表低分的陈述	High-Scoring Statement (Scored 7 at Endpoint) 代表高分的陈述
SF1	When problems arise in my life, I often worry that I won’t be able to solve them. 我经常担心我无法解决生活中的问题.	I always believe I can solve any problem that confronts me in life. 我总是相信我可以解决生活中遇到的任何问题.
SF2	I worry a lot about my ability to cope with life’s challenges. 我很担心无法很好地应对生活中的挑战.	I’m always very confident about my ability to do well in difficult life situations. 我总是自信我可以很好地应对逆境.
SF3	I am often fearful about my ability to solve problems. 我常常担心自己解决问题的能力.	I believe I can solve most of the problems I confront if I set my mind to them. 我认为只要我下定决心就一定能解决我遇到的大部分问题.
SF4	Compared with other people, I think I tend to struggle more with unexpected problems. 和大多数人相比, 解决意料之外的问题对我来说很艰难.	Compared with most people, I think I cope pretty well with unexpected problems. 和大多数人相比, 我能很好地解决意料之外的问题.
LoC1	I feel that what happens in my life depends a lot on chance and luck. 我觉得我生活中发生的一切主要取决于机会和运气.	I feel that what happens in my life is mostly up to me. 我觉得我生活中发生的一切主要取决于我.
LoC2	I feel like my life is mostly controlled by powerful others (e.g., parents, supervisors) 我感觉我的人生主要由那些更有权力的人掌控 (比如父母和领导).	I feel that my life depends mostly on my own actions and choices. 我感觉我的人生主要由我自己的行动和选择决定.
LoC3	I feel that whether I get what I want depends primarily on other people. 我觉得我能否得到我想要的东西主要取决于其他人.	I feel that I can get what I want no matter what others want for me. 我觉得无论他人如何我总是能够得到我想要的.
LoC4	If I fail at something, it will be because of bad luck or because others have interfered with me. 如果我在某件事上失败了, 那是因为运气不好或其他人干扰了我.	If I fail to achieve something, it will be because I didn’t try hard enough. 如果我没能做成某件事, 那是因为我没有尽力.
DwU1	I always feel anxious when the future is uncertain. 当未来充满不确定性时, 我总是感到焦虑.	The future is always uncertain; I don’t let it bother me. 未来总是充满不确定性, 我不会因此烦恼.
DwU2	Uncertainty frightens me. 不确定性让我感到害怕.	I think some uncertainty makes life exciting. 我认为不确定性让生活更刺激.
DwU3	I can’t stand not knowing what the future holds. 不知道未来会发生什么, 这让我无法忍受.	I see uncertainty as just a part of life. 我认为不确定性是生活中的一部分.
DwU4	Uncertainty really stresses me. 不确定性让我感到紧张.	I see an uncertain future as an opportunity to grow. 我认为不确定性对我来说是一个成长的契机.
M1	Usually, when I’m engaged in an activity, I often find myself thinking about other things. 当我做事的时候经常发现自己在想其他的事.	When I’m engaged in an activity, I always focus fully on what I am doing at that moment. 我做事的时候总是专注当下.
M2	I try to block out unpleasant thoughts when they occur. 我试图不去想不愉快的事情.	I allow whatever thoughts to occupy my mind, without thinking too much if they are pleasant or unpleasant. 我允许任何想法占据我的脑海, 无论它们是愉快的还是不愉快的, 我都不会想太多.
M3	I get angry when things don’t go the way I expect them to. 当事情没有按照我期望的那样发展时, 我会变得愤怒.	I find that unexpected events make life more interesting. 我发现意料之外的事情让生活更有趣.
M4	When something upsets me, I try to find a way to control my feelings. 当某些事情让我心烦意乱时, 我会试图找到方法来控制自己的情绪.	If something upsets me, I just accept that I’m upset without trying to control it. 如果某些事情让我心烦意乱, 我会接受我心烦意乱的状态而不试图控制它.

**Table 2:** EFA results with data from Group A (*n* = 414).

Item (Abbreviation)	Communalities	Rotated Factor Matrix			
	Extraction	1	2	3	4
Self-efficacy_1 (SF1)	0.88	0.87	0.23	0.23	0.17
Self-efficacy_2 (SF2)	0.88	0.85	0.26	0.25	0.19
Self-efficacy_3 (SF3)	0.78	0.77	0.26	0.27	0.20
Self-efficacy_4 (SF4)	0.70	0.69	0.33	0.26	0.24
Dealwithuncertainties2 (DwU2)	0.68	0.25	0.77	0.12	0.31
Dealwithuncertainties4 (DwU4)	0.58	0.27	0.74	0.29	0.25
Dealwithuncertainties1 (DwU1)	0.53	0.31	0.70	0.21	0.26
Dealwithuncertainties3 (DwU3)	0.62	0.26	0.67	0.34	0.19
Locusofcontrol2 (LoC2)	0.76	0.21	0.16	0.75	0.23
Locusofcontrol4 (LoC4)	0.77	0.20	0.17	0.71	0.10
Locusofcontrol3 (LoC3)	0.67	0.27	0.27	0.65	0.23
Locusofcontrol1 (LoC1)	0.70	0.23	0.21	0.57	0.32
Mindfulness 3 (M3)	0.72	0.21	0.48	0.16	0.64
Mindfulness 2 (M2)	0.50	0.15	0.10	0.25	0.64
Mindfulness 4 (M4)	0.49	0.12	0.25	0.16	0.62
Mindfulness 1 (M1)	0.54	0.29	0.34	0.21	0.55

**Table 3:** Correlation of factors.

	1	2	3	4
1. OverallSelf-Efficacy	1.00			
2. OverallLocus of Control	0.58 **	1.00		
3. OverallDealWithUncertainty	0.64 **	0.58 **	1.00	
4. OverallMindfulness	0.54 **	0.56 **	0.66 **	1.00

**: Correlation is significant at the 0.01 level (2-tailed).

**Table 4:** Fit indices for two models of the ISALES (Group B, *n* = 414).

Model	χ^2^	*df*	χ^2^/*df*	NNFI	GFI	CFI	SRMR
One-factor	1428.52	104	13.74	0.64	0.63	0.69	0.10
Four-factor	389.45	98	3.97	0.92	0.89	0.93	0.05

**Table 5:** Mean and standard deviation of internal strengths for two clusters.

Internal Strengths/ Mental Health Outcomes	Cluster 1 (*n* = 337)		Cluster 2 (*n* = 491)	
	Mean	SD	Mean	SD
Self-efficacy	6.02	0.73	4.23	1.26
Locus of control	5.92	0.66	4.38	0.99
Ability to deal with uncertainties	5.66	0.92	3.77	1.13
Mindfulness	5.62	0.94	3.86	0.86
WHO5_mean	3.49	1.06	2.84	1.02
DASS_Depression	1.41	0.43	1.71	0.59
DASS_Stress	1.62	0.55	1.99	0.63
DASS_Anxiety	1.40	0.46	1.56	0.54

## Data Availability

The data presented in this study are available on request from the corresponding author. The data are not publicly available due to ethical issues.
